# Long-lasting insecticidal nets in Zambia: a cross-sectional analysis of net integrity and insecticide content

**DOI:** 10.1186/s12936-015-0754-8

**Published:** 2015-06-10

**Authors:** Allen S. Craig, Mbanga Muleba, Stephen C. Smith, Cecilia Katebe-Sakala, Gershom Chongwe, Busiku Hamainza, Batuke Walusiku, Megan Tremblay, Maureen Oscadal, Robert Wirtz, Kathrine R. Tan

**Affiliations:** Centers for Disease Control and Prevention (CDC), Global Immunization Division, Atlanta, GA USA; Tropical Diseases Research Center, Ndola, Zambia; Centers for Disease Control and Prevention (CDC), Division of Parasitic Diseases and Malaria, Atlanta, GA USA; Bayer Ltd, Islando, South Africa; National Malaria Control Center, Lusaka, Zambia; Catholic Medical Mission Board, Lusaka, Zambia; Louisiana Department of Public Health, STD/HIV Program, New Orleans, LA USA; Neighborcare Health Pike Market Medical Clinic, Seattle, WA USA

**Keywords:** Insecticide-treated net, Malaria, Vector control

## Abstract

**Background:**

Long-lasting insecticidal nets (LLINs) are a mainstay of malaria prevention in Africa. More LLINs are available now than in any time previously due to increases in funding for malaria control. LLINs are expected to last three to five years before they need to be replaced. Reports of nets lasting less than three years are frequent in Zambia, which, if true, will increase the number of LLINs needed to maintain universal coverage.

**Methods:**

This study collected nets distributed during mass distribution campaigns. One net was collected from each participating home in 12 districts in 2010 and all nets were examined for holes. One household member was surveyed about net use and care.

**Results:**

The study collected 713 polyester nets with a median age of 31 months (range 27–44 months, interquartile (IQR) range: 29–36 months), median number of holes was 17 (IQR: 5–33), and median total hole size was 88.3 sq cm (IQR: 14.5-360.4). The median total number of holes did differ by age of the net, from 27–44 months, but not in a linear fashion. The difference in the number of holes in the newest and oldest nets was not statistically significant. The mean deltamethrin level for all nets was 23 mg/sq m (≥8 mg/sq m is considered effective). There was a larger total hole area in the lower half of the nets (repeat measures ANOVA, F = 228.43, df = 2, *p* < 0.0001) compared to the upper half and roof of the net. Only 8.7 % of nets had evidence of repairs.

**Conclusions:**

At 27 − 30 months, LLINs already had a large total hole surface area that was equivalent to the oldest nets observed. Nets were often tucked under reed mats which may explain the finding that the largest hole area was found in the lower half of the net. Studies need to be conducted prospectively to determine when physical deterioration occurs and why nets are discarded. Re-enforcing the lower half of the sides of LLINs may help decrease holes.

## Background

Long-lasting insecticidal nets (LLINs) are highly effective, essential components of worldwide efforts to control malaria [1–6]. The percentage of homes in sub-Saharan Africa owning at least one insecticide-treated net increased from 3 % in 2000 to 54 % in 2013 [7]. An estimated 298 million LLINs were distributed in sub-Saharan Africa from 2011 to 2013 at a total cost exceeding $1.5 billion, which fell short of the projected need of at least 150 million per year to protect all populations at risk [7]. In Zambia, the 2012 Malaria Indicator Survey found that 68 % of homes visited had at least one insecticide-treated net [8]. LLINs are typically made of polyester or polyethylene fibres treated with pyrethroid-class insecticides. Manufacturers state that LLINs will last three to five years with normal use or 20 washes, as determined by World Health Organization (WHO) Pesticide Evaluation Scheme (WHOPES). In many countries, including Zambia, there are anecdotal reports of nets lasting much shorter periods of time though replacements were planned per the net life suggested by the manufacturers. Given the large amount of money budgeted for LLINs, it is essential that the true life expectancy of LLINs in use by families in endemic countries is known in order to better quantify the optimal timing and quantities needed for LLIN replacement and to ensure that the population is protected by effective nets.

Many studies have looked at the physical integrity of nets and the persistence of insecticides after repeated washing [9–16]. WHO recently published guidelines for monitoring the durability of LLINs under field conditions [17, 18]. New hole-size measuring methods have been introduced to make field evaluations easier, including the use of common size standards [9, 17, 19, 20]. Much less is known about when an LLIN should be replaced because it is no longer effective. Rehman and colleagues noted that nets are less effective against malaria as holes become larger [21]. Allan *et al*. used a mean total net hole size of ≥1000 sq cm as the definition of an unserviceable net [14].

Zambia has distributed LLINs through mass campaigns and through antenatal clinics and under five years old clinics. A large scale-up has taken place over the past eight years, initially in an effort to protect high-risk individuals, such as pregnant women and children under five years of age and more recently to cover every sleeping space with an LLIN. Over 24 million LLINs were distributed from 2006 to 2011 [22]. The purpose of this study was to describe how physical integrity and level of insecticide varied with age among LLINs collected in four provinces in Zambia.

## Methods

### Study sites

This study was conducted in 12 districts in Eastern, Southern, Northern, and Copperbelt Provinces from February to October 2010. In the 2012 Malaria Indicator Survey, Eastern and Northern Provinces had parasite prevalence rates of 24-25 % while Southern and Copperbelt Provinces had prevalence rates of 5-8 % [8]. The primary vectors in these areas are *Anopheles funestus, Anopheles gambiae s.s.* and *Anopheles arabiensis*. The districts chosen within these Provinces were districts where the National Malaria Control Center (NMCC) or a partnering non-governmental organization (NGO) had distributed PermaNet® 2.0 LLINs between 2007 and 2008 via mass distribution campaigns.

### Study design

This was a cross-sectional study in which LLINs of various ages were collected and examined for physical integrity and persistence of insecticides. Any household that had received a PermaNet® 2.0 LLIN from the NMCC or its partnering NGO during mass distribution campaigns between 2007 and 2008 and was currently using the LLIN, was eligible to enrol. One LLIN was collected from each enrolled household, and replaced with a new PermaNet 2.0® donated by the manufacturer (Vestergaard-Frandsen). Upon enrolment, an interview was conducted with one adult age 18 years or older utilizing a brief questionnaire on care and use of the LLIN. LLINs were then examined for physical integrity by measuring holes and insecticide content with bromine x-ray fluorescence. LLINs of different net ages were then compared in terms of physical integrity and insecticide content.

### Sampling and sample size

The sample size was calculated taking into account a cross-sectional, two-stage, cluster sampling design (villages were randomly selected by the Ministry of Health or NGO staff and houses were selected randomly as detailed below). One of the outcomes of interest was the total area of holes in each net. There is no threshold total hole area that defines a failed net. So, an arbitrary surface area of 320 sq cm was chosen to reflect a hole with a diameter of 20 cm (approximately the size of a head) that would allow mosquitoes to enter a net. The formula used to calculate the diameter of rectangular holes was height x width = area. For this study to estimate differences in proportions of LLINs with a total hole area greater than 320 sq cm among LLINs of different net age groups, with a precision of 5 % and with a confidence level of 95 %, a sample size of 384 was needed. Since two-stage cluster sampling was done, this sample size was doubled (design effect = 2) to 768. A goal of 800 LLINs was established for this study.

From the list of the homes which received at least one LLIN, every other home (using a fixed interval of two) starting at the top of the list was offered enrolment until 50 were reached. Sixteen clusters were sampled. If a home was not willing to participate or was vacant at the time of the study team visit, the next home on the list was selected until 50 homes were enrolled. One LLIN was collected per study household. In all homes the LLIN that had been hanging the longest in the house was selected. In households with more than one LLIN hung at the same time, a random number was assigned to each LLIN and the LLIN with the highest number was selected, regardless of its physical condition.

### Questionnaire

One adult household member age 18 years old or older was interviewed with a short questionnaire. The questionnaire asked about household demographics, use and care of LLINs such as number of times washed during its lifetime, method of washing and drying of LLINs, and whether or not the LLIN was used the previous night. Questionnaires were translated from English into the local language by research assistants who were fluent in the language. If the research assistant did not speak the local language, the local volunteer accompanying the researcher was asked to interpret during the administration of the questionnaire.

### Evaluation of physical integrity

Each LLIN used in the study was stored in a plastic bag. The batch number and dates of production and hanging were recorded for each LLIN. Each LLIN was then evaluated on a 180-cm square frame made of plastic pipe with black plastic spread tightly over it to provide contrast for examining holes and tears. The height and width of each hole or tear was measured in centimetres and documented as to location (lower half, upper half or roof; all LLINs were rectangular).

### Determination of residual insecticide

A bromine X-ray fluorescence (XRF) analyzer was used to determine the bromine level as a surrogate for direct measurement of deltamethrin, the pesticide used in PermaNet 2.0® nets. An Innov-X Model XT-442 XRF (Innov-X Systems Inc, Woburn, MA, USA) was used to calculate bromine levels. It was calibrated using single-layer polyester net samples treated with known levels of deltamethrin. Its accuracy was further verified by analysing 150 samples of used LLINs, which were subsequently analysed using high-performance liquid chromatography (HPLC). The XRF analysis was performed on each net folded to provide 24 net surfaces for each XRF examination.

**Fig. 1:**
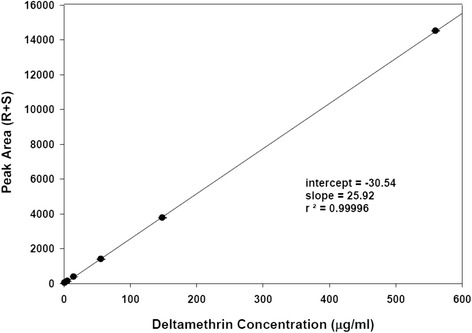
High-pressure liquid chromatography response using deltamethrin calibration samples of known concentration

To standardize this method, a 30 × 30 cm sample of 20 of the LLINs retrieved in the Zambia study had HPLC quantification of pesticide level conducted at the Centers for Disease Control and Prevention (CDC) in Atlanta. Calibration solutions of deltamethrin (99.9 %, Chem Service, Inc, West Chester, PA, USA) in 95/5 isooctane/1,4-dioxane were prepared at the following concentrations: 560.2, 148.6, 56.02, 14.86, 5.602, 1.486, and 1.120 μg/ml. Each solution was analysed five times. The results (Fig. 1) indicate an extremely linear response (R^2^ = 0.99996) over this concentration range. It is expected that the extracts from the Zambia samples would contain 0–20 μg/ml of deltamethrin and the response curve is clearly linear within and beyond this range. The limit of detection, calculated as three times the standard deviation of the results for the most dilute sample, was 3 × 0.04475 = 0.134 μg/ml. This corresponds to a concentration on 0.01 sq m of net of 0.670 mg/sq m.

A deltamethrin level of approximately 8 mg/sq m has been shown [19] to be the lower threshold of efficacy to achieve an 80 % mortality rate against susceptible anophelines; this was used as a cut-off point to dichotomize the deltamethrin levels into ‘effective’ (≥8 mg/sq m) and ‘ineffective’ (<8 mg/sq m) insecticide levels.

### Data analysis

The main outcome of interest was the sum of the surface area of all holes in each LLIN, which was referred to as the ‘total hole area’. The total hole area was determined for each net by summing the area of all individual holes calculated using the formula: area = πr^2^ (r = radius). The independent variables of interest were LLIN age (LLINs were grouped into six-month net age groups (25–30, 31–36, 37–42, and 43–48 months). LLINs without batch numbers or date of hanging were excluded because this information was used to determine the age of LLINs. Other variables of interest or potential confounders included province of residence, number of times a net was washed, and type of sleeping surface (reed mat or foam mattress).

Univariate analysis was done on continuous variables, and the median total hole area was calculated. The distribution of the values for total hole area was not normally distributed, and therefore, the Kruskal-Wallis one-way analysis of variance test, a non-parametric method using ranks to compare two independent samples, was implemented with Dunn post-hoc testing (a method to adjust for multiple comparisons which generalizes the Bonferroni adjustment, alpha = 0.05). The Kruskal-Willis analysis was used to compare total hole area by net age group.

To further examine the relationship between hole area and LLIN age, and to account for any factors that might confound this relationship or interact with the independent variable, analysis of variance (ANOVA) was done. The distribution of total hole area was examined, and deviances from normal distribution were corrected with a log transformation of total hole area. Significant independent variables were included in a full model, then using a backward stepwise procedure, a final adjusted model was obtained. To see what part of the net was more prone to developing holes, the repeated measure ANOVA was used to examine the relationship between total hole area and location on the net (roof, upper half, or lower half of the same LLIN), controlling for LLIN net age group.

To analyse data on insecticide content, the proportion of LLINs with ineffective deltamethrin levels was determined for each net age group. Factors that might be associated with insecticide content, such as total area of holes, age of LLIN and number of washes were also examined using bivariate analysis and multivariate logistic regression. Data were analysed using SAS v9.3 (SAS Institute Inc., Cary, NC, USA).

### Ethical considerations

Informed consent was obtained from all participants in their preferred language. The study was approved by both the Tropical Disease Research Center Ethics Committee and the CDC Institutional Review Board.

## Results

A total of 713 LLINs with ages confirmed by batch number and date of hanging were collected and included in the study. The median age of LLINs was 31 months (interquartile range (IQR) 29–36 months), most were used the night before (73.5 %), and the median number of washes was four (IQR 2–6). Almost all, 94 %, of the LLINs had holes. For all LLINs, the median total number of holes was 17 (IQR 5–33), and the median total hole area was 88.3 sq cm (min = 0, max = 19,241.9, IQR 14.5-360.4). Only 8.7 % of nets had evidence of repairs. A description of the LLINs by net age group can be found in Table 1.

**Table 1:** Characteristics of long-lasting insecticidal nets by age category of net

Characteristic	LLINs aged 25–30 months (*n* = 349)	LLINs aged 31–36 months (*n* = 193)	LLINs aged 37–42 months (*n* = 122)	LLINs aged 43–48 months (*n* = 49)
Any holes (%)	333 (95.4)	184 (95.3)	106 (86.9)	47 (95.9)
Median total number of holes (IQR range)	20 (8–39)	16 (7–32)	7 (2–21)	18 (8–32)
Median total hole area (IQR range)^a^	180.1 (28.4-645.9)^b^	106.5 (23.2-430.4)	30.2 (3.4-162.9)^c^	53.7 (11.5-221.4)^b^
Any seam failure (%)	101/348 (29.0)	42 (21.8)	20 (16.4)	15 (30.1)
Any burns (%)	188 (53.9)	108 (56.0)	51 (41.8)	20 (40.8)
Used last night (%)	283 (81.1)	159 (82.4)	64 (52.5)	18 (36.7)
Any repair (%)	37 (10.6)	16 (8.3)	7/120 (5.8)	2/49 (4.1)
Used above a reed mattress (%)	54/149 (36.2)	60/106 (56.6)	11/59 (18.6)	2/30 (6.7)
Ever washed	288/288 (100)	159/159 (100)	79/79 (100)	22/22 (100)
Median number of washes (IQR range)	4 (2–6)	3 (2–6)	3 (2–5)	4 (3−5)

^a^Significant differences among net age groups, Kruskal-Wallis Chi-square 43.4011, *p* <0.0001

^b^ Median total hole areas of oldest and youngest LLINs were not significantly different

^c^Significantly different from LLINs in the 24–30 months and 31–36 months net age groups by Dunn post-hoc testing

Values for total hole area were not normally distributed. Using non-parametric methods to compare total hole area by net age group, significant differences were found among these net age groups (Kruskal-Wallis Chi-square 33.6785, *p* <0.0001). Pair-wise comparisons with Dunn post-hoc testing revealed that the total hole areas of the youngest LLINs (aged 25–30 months) were not significantly different than those of the oldest LLINs (aged 43–48 months) (Table 1). Interestingly, the second to oldest net age group, LLINs 37–42 months old, had a median total hole area significantly lower than that of the two youngest net age groups.

To further examine the association between total hole area and net age group, ANOVA models were performed. The total hole variable was not normally distributed, so a log transformation of the variable was used. Unadjusted results showed a significant effect of net age group and log total hole area (F = 15.63, df = 3, *p* <0.001). When examining factors that might affect the relationship between net age group and total hole area, the province of residence seemed to be a significant factor in development of holes (F = 9.33, df = 3, *p* <0.0001). On closer inspection, one particular province had a significantly larger (*p* <0.001) proportion of older LLINs *versus* the other provinces, and was also the province with LLINs that had the largest total hole area. The final model adjusted for province because care behaviours not asked about in the survey could differ by province. Furthermore, having any burns on the LLIN and having any repairs was associated with having a larger total hole size (F = 59.21, df = 1, *p* <0.0001 and F = 15.93, df = 1, *p* <0.001). Because of the small number of LLINs that had any repairs (Table 1), this variable could not be included in the final model. It was also found that the number of washes, while not significantly associated with development of holes in terms of total hole area, interacted with the LLIN net age group. The older the LLINs, the more times the LLIN was washed. After adjusting for province of residence, having any burns on the LLIN, the number of times an LLIN was washed, and the interaction between number of washes and LLIN age, the final model showed that the log of total hole area did vary significantly with net age group (F = 8.68, df = 11, *p* <0.0001, Table 2). On closer examination, the adjusted mean of the log total hole size for nets aged 25–30 months, 31–36 months, 37–42 months, and 43–48 months, were 4.69, 4.26, 2.97, and 4.2 respectively; LLINs aged 37–42 months had significantly lower log total hole size than other age groups. No significant associations were observed between the log of the total hole size and other factors such as having slept under the LLIN the night before, using a reed mat, having higher than elementary school education, cooking in the same room as the LLIN, drying the LLIN outside, or beating the net on rocks while washing it (a rare occurrence, less than 0.6 %).

**Table 2:** Final model of log of total hole area and net age group (*N* = 713)

Variable	df	F-value	*p*-value
Net age group	3	4.64	0.0032
Having any burns on LLIN	1	39.82	<0.0001
Province of residence	3	0.34	0.7960
Washing LLIN >4 times	1	2.92	0.0879
Interaction of net age group and having washed LLIN >4 times	3	2.99	0.0304

The total hole area was also found to change depending on where on the net the holes were located: roof, upper half, or lower half of the LLIN (repeat measures ANOVA, F = 249.0, df = 2, *p* <0.0001). The median total hole area by location on the LLIN is shown in Table 3. There was a larger median total hole area in the lower half of the LLIN than the upper half and roof. The use of any particular sleeping surface was not associated with development of holes in the lower half of the LLIN.

**Table 3:** Median total hole area at different locations on the long-lasting insecticidal net

LLIN location	Median total hole area sq cm^a^ (IQR range)
Roof	0 (0–5.9)
Upper half	13.1 (1.26-86.6)
Lower half	34.5 (4.1-179.6)

^a^Significantly different by repeat measures ANOVA, *p* < 0.00

The overall number of LLINs having less than the minimum threshold for insecticide activity, 8 mg/sq m deltamethrin, was 157 (22.0 %). The proportion of LLINs aged 25–30, 31–36, 37–42, and 43–48 months with deltamethrin content less than 8 mg/sq m were 22.6, 20.7, 15.6, and 38.9 %, respectively, and were significantly different (*p* = 0.01). LLINs washed more than four times were significantly more likely to have an insecticide content lower than the threshold (unadjusted OR 1.9, 95 % CI 1.2-2.9). When controlling for net age group, number of washes > four was significantly associated with a deltamethrin content of <8 mg/sq m. Other factors found to not be associated with insecticide content were total hole area, use of an LLIN the previous night, having any burns on the LLIN, having any repairs, the LLIN having been washed on rocks, the type of sleeping surface used, and cooking location.

## Discussion

This cross-sectional study of over 700 polyester nets age 27–44 months old did not find that total hole area increased with age, as was expected. There was no significant difference in the log of total hole area between the oldest and youngest LLINs. Rather, it was noted that LLINs 37–42 months old, had a median total hole area significantly lower than that of the two youngest aged nets. This is likely due to early attrition of nets that become severely damaged and are either discarded, given away or used for something other than preventing mosquito bites at night [14, 18, 23]. Because a net may be taken out of service by family members at any time during its lifetime, more information is needed on the reasons for net attrition over time. The 37-42-months old nets also had the lowest number of nets with <8 mg/sq m deltamethrin levels, which is more difficult to explain because they were washed as frequently as the other nets and a similar proportion of nets in each net age group were dried in the sun. It is also possible the 37-42-months old nets endured a particularly dry season and where not used as often as other nets in the study.

Not surprisingly, this study showed that the total hole area was significantly higher in the lower half of these rectangular nets compared to the upper half or the roof. There was no difference in the total size of holes when reed mats were compared to mattresses. Newer versions of Permanets® and Netprotect® nets have reinforced the lower portion of their nets with the intention of preventing development of holes in this area of the LLIN, but a field evaluation of these LLINs is needed to see if this is indeed the case.

The finding that washing nets more than the median of four times correlates with deltamethrin levels <8 mg/sq m is surprising given the WHOPES requirement that nets maintain adequate insecticide levels up to 20 washes over the life of each net [17]. It is likely that net owners underestimated the number of times each net was washed which is typically two or more times per year [19, 24]. The method of washing or drying, or the location of cooking (inside or outside the house) did not appear to impact deltamethrin levels. Use of X-ray fluorescence in the field appears to be a useful and accurate tool for estimating deltamethrin levels [10].

This study did not attempt to define net failure, that is, when a net no longer protects its inhabitants. Others have suggested that a proportionate hole index of >276 allows mosquitoes access to persons sleeping under nets [25] or that nets with a proportionate hole index of >300 (the equivalent of 1000 sq cm total hole area) may no longer be useful [14]. Although scientists may ultimately agree on what constitutes a ‘failed net’ it is as important to learn when net users decide to stop using a net [24]. Longitudinal studies will help shed light on this [17].

Other measurements of holes have been suggested, such as the finger, fist and head method which are field-friendly. Furthermore, a proportionate hole index (PHI) has also been suggested, which involves dividing hole surface area by the smallest hole to arrive at a unitless index. In order to make the results as comparable as possible and because the study was designed before the use of PHIs, total hole area in sq cms was chosen for the outcome of interest.

Limitations to this study include its cross-sectional design which does not allow conclusions to be made on the causes of holes, and the true association of LLIN age and hole development, as could be done in a longitudinal study. This approach also did not allow the assessment of net attrition as recommended by more recent WHO guidelines [17]. The data do, however, help describe a snapshot of what is happening in the field to these LLINs, and can assist with hypothesis generation. LLIN repair was not common in the study nets, but may have the potential to affect LLIN longevity and should be studied further. Because there is no definition of LLIN failure, the sample size was calculated using an artificial threshold for total hole area that would constitute a failed LLIN. The number of nets with low deltamethrin levels associated with a relatively low number of reported washes likely reflects inaccurate recall about the number of times a net was washed over the years of use. Households chosen for enrollment were selected from a list using a fixed interval of two and substitution of households was allowed when a home did not have a net or no one was present during the visit of the research team, which could have introduced bias. Also, only one net was studied per household regardless of the number of nets present, in order to avoid households with more nets biasing the study by being over-represented.

## Conclusions

This study showed a lack of increase in total hole area as nets aged. This is likely due to LLIN attrition that might occur between two and three-and-a-half years. Longitudinal studies are needed to determine the attrition rate and understand reasons for LLIN loss. The lower half of these rectangular nets had the most physical deterioration compared to the other parts of the LLIN, making reinforcement of this portion of a net potentially useful. Studies of the durability of newer LLINs with the reinforced lower part of the net should be done.
